# Effect of Age and Dietary Intervention on Discrimination Learning in Pet Dogs

**DOI:** 10.3389/fpsyg.2018.02217

**Published:** 2018-11-14

**Authors:** Durga Chapagain, Zsófia Virányi, Ludwig Huber, Jessica Serra, Julia Schoesswender, Friederike Range

**Affiliations:** ^1^Clever Dog Lab, Comparative Cognition, Messerli Research Institute, University of Veterinary Medicine Vienna, Medical University of Vienna, University of Vienna, Vienna, Austria; ^2^Royal Canin Research Centre, Aimargues, France; ^3^Domestication Lab, Konrad Lorenz Institute of Ethology, University of Veterinary Medicine Vienna, Vienna, Austria

**Keywords:** aging, dog, picture discrimination, learning, dietary intervention

## Abstract

Aging is associated with a decline in cognitive functions such as learning, memory, attention, cognitive flexibility, and executive functions. Recent evidence indicates that interventions such as exercise, diet and cognitive training can be used to reduce the rate of age-dependent cognitive decline. In this study, we examined the changes in discrimination learning in older pet dogs, tested whether a dietary intervention counteracts a potential decline in learning and evaluated the influence of lifelong training on learning speed and cognitive flexibility. We included 115 pet dogs (>6 years) of 30 different breeds into one of two treatment groups: either a diet enriched with antioxidants, docosahexaenoic acid (DHA), Phosphatidylserine and tryptophan or a control diet for 1 year. Lifelong training was calculated for each dog using a questionnaire where owners filled their dog’s training experiences over years. Dogs were trained to discriminate different pictures at the start of the dietary intervention using a touch screen methodology. After 1 year of dietary intervention, they were tested on a main picture discrimination task where they were confronted with a discrimination of four new pictures. We used the total number of sessions needed to reach learning criterion as a measure of learning speed and the rate of correction trials as a measure of deficit in learning from feedback/cognitive flexibility. In the main discrimination task, we found an influence of neither age nor diet on the speed of learning and deficit in learning from feedback. We did not find any influence of lifelong training either. The null findings were further corroborated by Bayesian statistics. The null findings might be due to the fact that pet dogs live in a stimulating environment which may reduce the rate of cognitive decline and hinder finding an age or diet effect. Also, the similarity between the training and the main discrimination task might have made the main task too easy for the animals to solve. Further studies are warranted to assess the effect of enriched diets on pet dogs using tasks that measure cognitive functions with a higher sensitivity.

## Introduction

Aging has been shown to be associated with a decline in cognitive functions such as learning, memory, attention, cognitive flexibility, and executive functions in humans (Boutet et al., 2005; Weiler et al., 2008), monkeys (Voytko, 1999), rats (Barense et al., 2002; Schoenbaum et al., 2002; Brushfield et al., 2008), and dogs (Adams et al., 2000). Recent evidence indicates that interventions such as physical exercise, diet and cognitive training can reduce the speed of age-dependent cognitive decline (Deary et al., 2009; Davis and Head, 2014). Physical exercise and cognitive training induce both temporary and permanent changes at the structural and functional levels in the aging brain thereby promoting physical and cognitive health of older humans (Churchill et al., 2002; Colcombe et al., 2003; Chang et al., 2012; Shatil, 2013; Bamidis et al., 2014; Berchicci et al., 2014; Bullock and Giesbrecht, 2014). Similarly, dietary manipulations are thought to enhance cognitive abilities by protecting the brain from oxidative damage, promoting repair and counteracting the effects of aging (Gómez-Pinilla, 2008). For example, reduction of oxidative damage and Aβ plaque pathology in the brain as well as reduced mitochondrial dysfunction have been reported in laboratory dogs fed with diets enriched with antioxidants and mitochondrial enzymatic co-factors (Milgram et al., 2005; Opii et al., 2008; Head et al., 2009; Pop et al., 2010). In line with these results, aged rats and laboratory dogs receiving an antioxidant treatment, as compared to subjects receiving a control diet, showed improved cognitive performance in the Morris water maze and in discrimination learning tasks (Socci et al., 1995; Davis and Head, 2014). Moreover, supplementation of medium chain triglycerides had beneficial effects on the aging brain, as demonstrated by better performance of laboratory dogs in various discrimination tasks (Pan et al., 2010). Finally, a combined treatment of behavioral enrichment (consisting of exercise and cognitive training) and antioxidants diet is thought to have additive effects on synaptic plasticity and cognitive functions in humans and in animal models (Cotman and Berchtold, 2002; Gómez-Pinilla, 2008), and at least in laboratory dogs helps attenuate age-dependent cognitive decline (Cotman et al., 2002; Milgram et al., 2004, 2005; Head et al., 2009). Overall, studies in humans, rats and dogs have provided some evidence of protective effects of diet and exercise on various cognitive functions in the elderly.

Various tests have been developed in humans, non-human primates, rats and dogs to measure cognitive decline due to aging (van der Staay et al., 1990; Herndon et al., 1997; Chan et al., 2002; Milgram et al., 2002a; Tapp et al., 2003a; Zeamer et al., 2011; Snigdha et al., 2012; Staub et al., 2013; Joly et al., 2014; Woods et al., 2015; Wallis et al., 2016; Chapagain et al., 2017). These tests show decline in learning, memory, decreased attention span, increased reaction time and deficits in inhibitory functioning in older humans and animals. Among different tasks used in the studies, discrimination and reversal learning are considered as the most widely used paradigms that are mostly used to measure changes in learning and cognitive flexibility during aging. Discrimination learning tasks generally utilize a two-choice procedure, where two stimuli are presented simultaneously and only the selection of the target stimulus leads to a reward (Mell et al., 2005). This means that the subject is required to attend to a target stimulus, while ignoring or avoiding distracting information (Julesz and Schumer, 1981). Simultaneous processing of stimuli declines during aging in humans and dogs, which is due to a decrease in processing speed, reduced cognitive resources and an inability to ignore distracting information (Lavie, 1995; Baddeley et al., 2001; Costello et al., 2010; Snigdha et al., 2012). These age-related impairments in discrimination learning are usually shown by an increase in the number of trials needed to reach a learning criterion (learning speed) and an increase in the rate of correction trials (cognitive flexibility) (Milgram et al., 2002b; Tapp et al., 2003b; Snigdha et al., 2012).

So far, object discrimination, size discrimination, black/white discrimination, landmark discrimination and oddity discrimination tasks have been utilized in laboratory dogs (for a review, see Davis and Head, 2014), while picture discrimination tasks have been used in pet dogs (Wallis et al., 2016). Wallis et al. (2016) studied 95 pet Border collies (aged from 5 months to 13 years) divided into five age groups in a picture discrimination learning task to examine age-related changes in learning abilities. The study reported a steady decline in learning speed as dogs grew older. When looking at the age groups, dogs aged from 5 months to 1 year took the lowest number of sessions to reach the learning criterion compared to all other age groups, highlighting that this age group was already performing at the peak level in discrimination learning. All dogs over 3 years took more sessions to reach criterion, demonstrating that their learning abilities begin to decline early during life. Furthermore, also the measure of deficit in learning from feedback showed linear increase with age, with oldest dogs being mostly affected. Therefore, the discrimination learning tests used by Wallis et al. (2016) has proved suitable to detect age effects. Discrimination learning tasks have also been used to test the influence of dietary supplements on learning and cognitive flexibility in laboratory dogs (Milgram et al., 2002a,b, 2004, 2005). In a longitudinal study using laboratory beagles, Milgram et al. (2002a) tested the effectiveness of antioxidants and behavioral enrichment in different cognitive tasks. Older laboratory dogs (>8 years) on antioxidant diet committed fewer errors compared to dogs on a control diet in a landmark discrimination (Milgram et al., 2002a), size discrimination (Milgram et al., 2004) and oddity discrimination (Milgram et al., 2002b). Moreover, with an antioxidant treatment, visual discrimination improved and reversal learning ability was maintained over time while untreated animals showed a progressive decline (Milgram et al., 2005). The results of these studies highlight the beneficial effects of supplementing antioxidants in aged dogs’ diet.

The aging process is associated with a progressive accumulation of oxidative damage that could play a role in the development or accumulation of neuropathology typically observed in age-related neurodegenerative disorders. The use of antioxidants such as vitamin E, vitamin C, beta-carotene, flavonoids, and polyphenols are shown to reduce the level of oxidative damage and delay or reduce age related cognitive decline (Opii et al., 2008). Nutritional antioxidants act as free radical scavengers by directly neutralizing free radicals, or reducing the peroxide concentrations and repairing oxidized membranes (Fusco et al., 2007). Similarly, natural phospholipids in the form of Phosphatidylserine act as a neuro-protector that helps in protecting the neurons against degenerative processes during aging. Phosphatidylserine has been documented to improve cognitive functions, such as memory, orientation, learning, and social behavior (Osella et al., 2008). Dietary supplementation with docosahexaenoic acid (DHA) has been found to elevate levels of hippocampal brain-derived neurotrophic factor (BDNF) and enhance cognitive function in rodent models of brain trauma. DHA might enhance cognitive abilities by facilitating synaptic plasticity and/or enhancing synaptic membrane fluidity. Moreover, it might also act through its effects on metabolism, as DHA stimulates glucose utilization and mitochondrial function by reducing oxidative stress (see review by Gómez-Pinilla, 2008). Observational epidemiological data in humans suggest that increased fatty fish and n-3 LCP consumption is associated with reduced risk of impaired cognitive function (Kalmijn et al., 2004). In the current study, we fed the animals with a diet enriched in antioxidants (vitamin C, vitamin E, and polyphenols), DHA, phosphatidylserine and tryptophan, and tested whether this nutrient cocktail shows any effect on aged dog’s learning speed or flexibility in learning from feedback. Since we cannot disentangle single ingredient from this nutrient cocktail, based on the available evidence from different studies, we suggest that the combination of the ingredients in this nutrient cocktail would improve cognition.

Therefore, our objectives in this study were to examine the changes in discrimination learning in older pet dogs of various breeds and to test whether a dietary intervention can counteract potential age-associated decline in learning and learning from feedback (cognitive flexibility) while taking also the dogs’ lifelong training experiences into account. In contrast with other studies in pet dogs (Dodd et al., 2003; Heath et al., 2007) that studied the effect of dietary supplements over shorter periods (42 and 60 days) and measured changes in dog behavior by means of questionnaires filled in by the owners, we fed the dogs with two kinds of diet for 1 year and used objective measures of changes in learning. In a recent study using 185 pet dogs aged over 6 years (Chapagain et al., 2017), we have found that lifelong training had a positive effect on sustained and selective attention probably due to the stimulation provided by these interactions. Therefore, in the current study, we hypothesized that training and diet interventions will reduce potential learning deficits, and predicted that (1) older dogs in both diet groups will be slower in learning and show a greater deficit in feedback from learning than younger dogs (Milgram et al., 2002b; Tapp et al., 2003b; Snigdha et al., 2012; Wallis et al., 2016), and (2) dogs on an enriched diet will perform better in discrimination learning task as compared to dogs fed with a control diet (Cotman et al., 2002; Milgram et al., 2002a, 2005; Pan et al., 2010), (3) dogs with a higher lifelong training score will perform better in our discrimination learning task compared to dogs with a low training score or no training.

## Materials and Methods

### Ethics Statement

The institutional ethics and animal welfare committee at the University of Veterinary Medicine, Vienna (Protocol number: 05/03/97/2014) approved this study. All dog owners signed a consent form at the start of the study.

### Animals

One hundred and fifteen pet dogs of 30 different breeds including mixed breeds were enrolled in the study. The dogs’ age ranged from 6.2 years to 14.2 years (74–170 months). Their mean weight was 22 kg (range: 7–42 kg). The average life span of dogs included in the study was 11–12 years (reference for life span of each breed was taken from American Kennel Club^1^). Prior to inclusion in the study, all dogs were thoroughly examined by a veterinarian and had a standard complete blood cell and serum biochemistry performed to ensure that they were healthy and eligible to participate. Dogs with serious mobility problems and severe loss of visual capacity were not included. During recruitment, owners filled in an extensive demographic questionnaire detailing their dog’s lifelong training experiences on 13 different types of training [Puppy school, obedience, agility, BGH (Begleithund), protection dog training, service dog training, search and rescue training, dog dancing/trick training, dummy training, hunting/nose work, sheep dog training, therapy dog training, others]. For each type of training a lifelong training score was calculated for each dog based on their past and current training attendance as follows: no experience = 0, sporadic training = 1, once or twice a month = 2, once or twice a week = 3 and completed training (with or without an exam) = 4. Lifelong training score was obtained by summing up all the collected scores and could range from 0 to 52. The average lifelong training score of the dogs was 11.64 (range = 0–34).

### Dietary Intervention

The 115 dogs were divided into two groups matched for age, sex, lifelong training score, weight and breed, and each group received either the control or test diet for a period of 1 year. The control and the test diets were similar in composition except that the test diet was also enriched in antioxidants, DHA, phosphatidylserine, and had a higher level of tryptophan. The diets were manufactured by a private pet food company and supplied to Clever Dog Lab, Vienna. The exact composition of the diets cannot be revealed because it is protected by a confidential clause. The caloric density of both diets was 3,864 kcal/kg. Nutritive intake of each dog was calculated separately based on the age, weight and body condition score. Dog owners were given food bags every month and instructed to exclusively feed their dogs with the given diet, with no more than 10% of other treats. On the days when the dogs came for training at Clever Dog Lab, dogs got low calorie training treats and did not receive any other treats from their owners. The food bags received from the dog food company were labeled either diet 1 or diet 2 and neither the experimenter nor the owner knew which were the control and the enriched diet until all analyses have been conducted.

### General Set-Up of the Experiment

After the introduction of test and control diets, dogs (*n* = 115) started with the experiment. It was divided into three training steps and one main discrimination task. The aim of the training steps (phases 1–3) was to familiarize the dogs with the contingencies of the 2-choice discrimination task in a stepwise manner. To pass from one phase to the next, dogs had to reach specific learning criterion (see below). The main discrimination task, conducted once a dog had received the diet for 1 year, was similar to the discrimination task in phase 3, but we used new stimuli rendering it a new learning task.

### Apparatus

**FIGURE 1:**
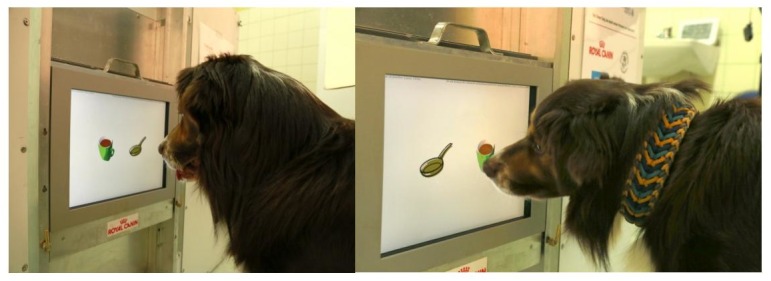
Photograph of a dog working on the testing niche of the touchscreen apparatus.

**FIGURE 2:**
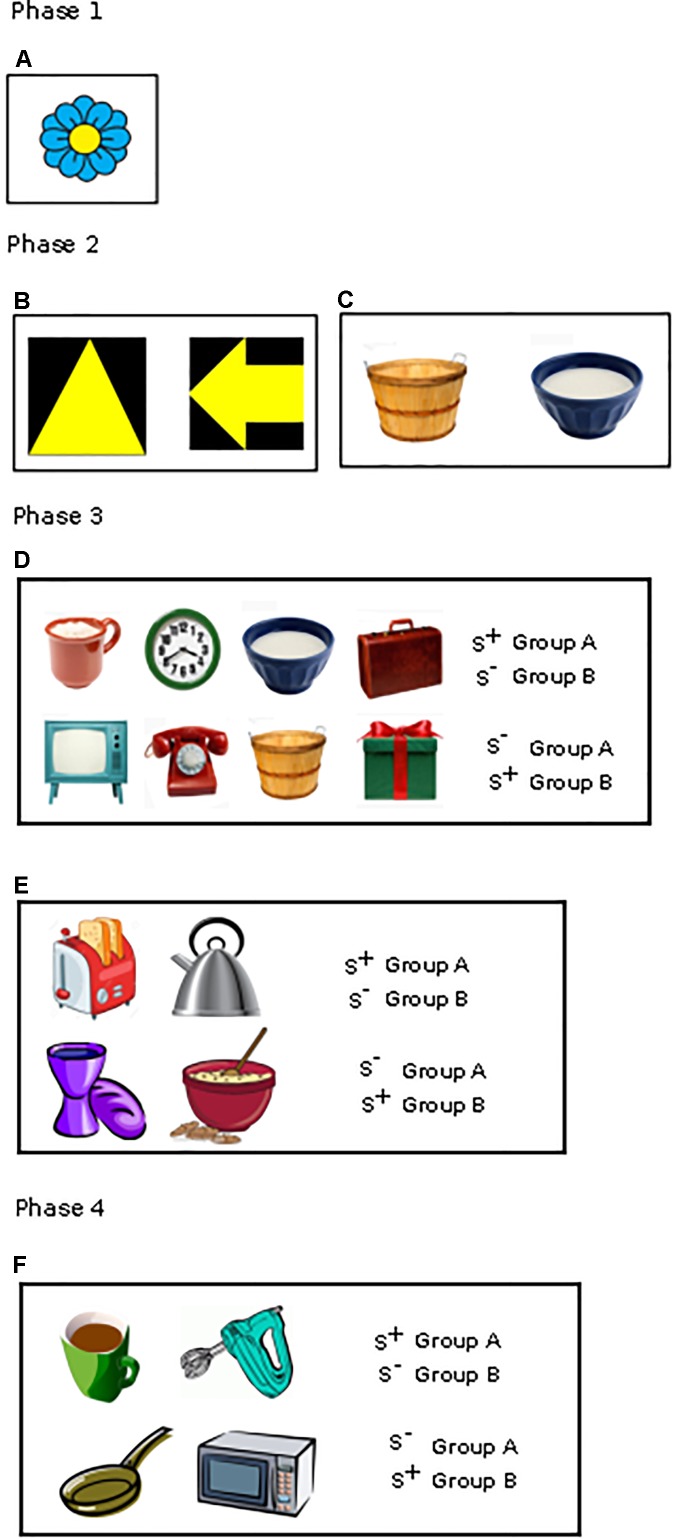
Different stimuli used in the various phases: **(A)** Approach training stimuli (phase 1), **(B,C)** Pre-training stimuli (phase 2): Geometric form **(B)** and Picture discrimination task **(C)**, **(D,E)** Picture discrimination (phase 3): 4 vs. 4 stimuli **(D)**, 2 vs. 2 stimuli **(E)**, **(F)** Main discrimination task (phase 4).

The touchscreen apparatus consisted of a 15” TFT computer monitor that was mounted behind an infrared touch frame and a feeding device that distributed treats (Figure 1 and Supplementary Figure S1). The monitor and touch frame could be slid up and down to be adjusted to the height of the dog. The height was set so that the center of the screen was located at the dog’s eye level. Movable screens were located at the front of the apparatus, which could be folded out to create a ‘testing niche’ and helped to prevent distraction from the external environment, and also served to position the dog in the ideal location to utilize the touchscreen. The stimuli displayed on the touchscreen consisted of jpeg clip art images obtained from the Internet presented on a white background. The stimuli differed in color, global outline, and internal features (Figure 2). For aged dogs we found the optimum size of the stimulus to be 200 by 200 pixels, which is equivalent to about 5 cm in size. If a dog touched the stimulus with its nose, the infrared light grid was interrupted, which triggered an acoustic signal and delivery of a food treat.

### Training and Testing Procedure

Dogs needed to touch the positive discriminative stimulus on the screen with their nose to get the reward (Figure 1). Rewards were administered in the form of small commercial dog food pellets (a low calorie training treat cut into two pieces) that were made available through a small hole beneath the touchscreen apparatus or the treat and train automatic food dispenser. Owners were instructed not to feed their dogs for at least 3 h before coming to the Clever Dog Lab to keep their motivation for working on the touchscreen high. During all three phases of the training as well as during the main discrimination task, the dogs visited the Clever Dog Lab at least once a week and participated in two to four training sessions over a half an hour period with short breaks in between sessions. Each session consisted of 30 or 32 trials (see below).

#### Phase 1: Approach Training on the Touchscreen Apparatus

All dogs (*n* = 115) were trained to touch the screen of the touchscreen apparatus with their nose using a clicker-aided shaping procedure or luring with food treats. They also learnt to use the food delivery system during this phase (3–18 sessions). A stimulus, clip art of a flower (Figure 2A), appeared in random locations on the white screen of the touchscreen apparatus. Dogs needed to touch that stimulus with their nose to get the food reward. The correct response was recorded when the dog precisely touched on the flower whereas in an incorrect response it touched anywhere but the flower. Since this is the first phase where the dog is still learning the task, if the dog took longer time to touch the flower, then the experimenter repeatedly encouraged the dog to touch the flower. If the dog refused to work on the touch screen despite repeated encouragement, then the session was terminated. The learning criterion was set until the dogs precisely touched on the stimulus and attained ≥28 correct choices in 30 trials (93.33%) in three consecutive sessions.

#### Discrimination Tasks

Once they completed Phase 1, they moved on a series of discrimination tasks using a forced two choice procedure where one positive discriminative stimulus (S+) and one negative discriminative stimulus (S-) were presented simultaneously on the left and right side on the touch screen (Figure 1) with the sides randomly changing from trial to trial (left/right). When the dog touched the positive discriminative stimulus (S+), the stimuli disappeared, a short tone was emitted by the computer, and a food reward was provided, ending the trial. The next trial was presented after 2 s of presenting empty white background. If the dog touched the wrong stimulus (S-), the stimuli disappeared, a short buzz sound was produced, and the screen turned red for 3 s. In this case, a correction trial was immediately initiated: the stimuli of the previous trial were presented again in the same positions. Correction trials were repeated until the dog responded correctly.

During all phases of discrimination tasks, the owner and researcher were both present in the room. They were seated on chairs at a distance of 100-150 cm from the dog and touchscreen apparatus, both were passive and did not interact with the dog when the dog was working on the touchscreen. In some cases, if the dog needed motivation to touch the screen, then the researcher or the owner could verbally encourage the dog to approach and touch on the screen or point toward the apparatus. After the session was finished, the dog was given a break and the owner could talk with the dog before the next session was started.

##### Phase 2: Discrimination pre-training

The aim of this training phase was to make the dogs familiar with the discrimination task in the touchscreen, starting with two pictures (1 vs. 1). The recruitment of 115 dogs was done in four periods, and therefore, we trained the first 45 recruited dogs in a geometric form discrimination task (arrow and triangle, Figure 2B), but during the training period we realized that the dogs had *a priori* preference for either of the shapes so that some dogs finished the training very fast while some took a very long time. Therefore, for our second, third and fourth cohort of recruited dogs (*N* = 70), we changed the methodology and used clip arts to train the next 70 dogs to discriminate two stimuli (Figure 2C; for more information see Supplementary Material). For all, the learning criterion was set at ≥20 correct first choices in 30 trials (66.66%) in four out of five consecutive sessions. We set this learning criterion since this was just significant above the chance level. Stimuli and procedure are described in details in the Supplementary Material.

##### Phase 3: Discrimination training

Dogs (*n* = 106) had then their training pursued with a higher number of pictures (Figures 2D,E). Dogs in each diet group were split into two stimuli groups (Group A and Group B) balanced for age and sex with opposite reward contingencies. Thirty-four dogs (16 dogs on test diet and 18 dogs on control diet) had to learn to discriminate eight pictures (4 vs. 4, Figure 2D) while 72 dogs (37 dogs on test diet and 35 dogs on control diet) received the same task with only four pictures (2 vs. 2, Figure 2E). Due to the high failure rate of the dogs in the initial cohort tested in the eight-picture discrimination task, we decided to reduce the number of pictures to four for the dogs that were recruited later. The data of these two groups of dogs were treated separately. The four or eight pictures were all different clipart. The learning criterion was set at ≥26 correct first choices in 32 trials (81.25%) in two consecutive sessions in the 4 vs. 4 discrimination task and ≥27 correct first choices in 32 trials (84.3%) in two consecutive sessions in the 2 vs. 2 discrimination task. The learning criterion was set based on the difficulty of the task.

##### Phase 4: Main discrimination task

After 1 year on the respective diets, 79 dogs (see Supplementary Table S1) participated in the main discrimination task with four *new* clipart pictures (Figure 2F). Out of the 79 dogs, 74 had reached the learning criterion in phase 3, while for four dogs that had worked on phase 3 training for 1 year without reaching the criterion albeit close (126, 88, 65, 57 sessions), training was stopped, and the dogs were given a break of 3 months. Afterward, these dogs were also tested on the main task. Finally, one dog who dropped from phase 3 training due to health issues also participated in the main discrimination task. Dogs in each diet group were split into two stimuli groups (Group A and Group B) balanced for age and sex with opposite reward contingencies. The presentation of the new stimuli, the training procedure and the learning criterion were the same as during the 2 vs. 2 discrimination training of phase 3. Diet was continued throughout the main discrimination task.

### Data Analyses

All statistical analyses were performed in R 3.2.2 (RStudio 2016) (R Core Team, 2016) and the graphical illustrations were done in IBM SPSS statistics Version 24. Data from the approach training and pre-training were not analyzed since dogs were sometimes helped by the experimenter in these phases (see Supplementary Material), which might have influenced the outcome. As dependent variables, we used (1) total number of sessions needed to reach learning criterion as a measure of learning speed and (2) rate of correction trials (total number of correction trials/total number of trials) as a measure of deficit in learning from feedback. We calculated the rate of correction trials to correct for the different number of sessions each animal needed to reach criterion. The cumulative correction trials were used as a dependent variable since they indicate cognitive flexibility in the sense that the animals with a lower number of correction trials, are able to switch more easily from the incorrect choice to the correct choice showing a higher cognitive flexibility. Rate of correction trials was not correlated with the total number of sessions to reach learning criterion. Therefore, we used two dependent variables in the analysis. For total number of sessions to reach criteria, we used generalized linear models (Poisson regression) and, to correct for overdispersion, a model with negative binomial error structure (R package “MASS” (Venables and Ripley, 2002) was used. Since we had count data for the number of sessions to reach criteria, we calculated the rate ratio for determining the effect size measure of predictors. The standard effect size measure for Poisson family regression models is the rate ratio (RR). Since this is a non-linear model, the effects are interpreted differently from typical linear models like ANOVA and linear regression. Here the effect is multiplicative, i.e., for a one unit increase in the predictor; we expect the predicted count to be multiplied by e^b1^ (Coxe et al., 2009).

We used Shiny app developed by Dr. Stefany Coxe^2^ to calculate the effect size measure. Interpretation of rate ratio: Effect size = 1 indicates no effect. Values greater than 1 indicate that the outcome mean when predictor = 1 is higher than the outcome mean when predictor = 0. Values less than 1 indicate that the outcome mean when predictor = 1 is lower than the outcome mean when predictor = 0. For the analysis of the rate of correction trials, we used linear models. Normality and homoscedasticity were assessed via residual distribution charts and plots of residuals against fitted values. In order to determine effect size [partial eta squared ($\eta_{p}^{2}$)] of predictors used in the model, we used R package “sjstats” (Lüdecke, 2018). According to Cohen (1969, pp. 278-280), Richardson (2011) provides partial eta squared values of 0.0099, 0.0588, and 0.1379 as benchmarks for small, medium, and large effect sizes, respectively. All the results are presented as mean ± SD unless otherwise indicated.

In addition to analysing the data using generalized linear models and general linear models, we also explored the effect of hypothesis guessing using Bayesian statistics for phase 3 discrimination training and the main discrimination task data. Hence, the results are supplemented with Bayes Factors (BF). To calculate BF, we used R package “rstanarm” (Stan Development Team, 2016) and “bridgesampling” (Gronau and Singmann, 2017). The package “bridgesampling” helps to calculate BF by comparing two models, one with the predictor of interest and another model without the predictor.

The central question of our experiment was to determine whether learning and cognitive flexibility differed between the two diet groups (hypothesis H1). In contrast, the null hypothesis (H0) would assume no difference between the two diet groups. Frequentist inference statistics only allows testing H1-hypotheses by rejecting the null-hypothesis. However, if the null-hypothesis cannot be rejected, it does not mean that it is true. Bayesian statistics (Dienes, 2014) allow determining whether the data provide stronger evidence for H1 or the null-hypothesis. The Bayes factor B means that the data are B times more likely under the H1 than under the null-hypothesis. If the BF is larger than 1, support for the H1 is stronger, whereas a BF smaller than 1 means that support for the H0 is stronger. A BF > 3 can be interpreted as moderate evidence, whereas a BF > 10 is considered strong evidence for accepting H1.

#### By Analyses

##### Total number of sessions to reach learning criterion

Phase 3: Discrimination training

Data for 4 vs. 4 picture discrimination and 2 vs. 2 picture discrimination tasks were analyzed separately as dogs took more than twice as many sessions to reach the learning criterion (48.9 ± 21.01 sessions vs. 18.6 ± 12.97 sessions). In the model for analyzing 4 vs. 4 picture discrimination data (*n* = 26), we included diet (test diet or control diet), age in months, lifelong training score as an independent variable and the interaction of age in months and diet to test whether age effects differed between the diet groups. In the model for analyzing 2 vs. 2 picture discrimination data (*n* = 61), we included diet (test diet or control diet), age in months and lifelong training score as an independent variable, and the interaction of age in months and diet to test whether age effects differed between the diet groups. We also included stimuli (Group A or Group B) and interaction of diet and stimuli as control factors.

Phase 4: Main discrimination task

From the 71 dogs that reached the learning criterion, 3 dogs had to be excluded from the analyses as their owners fed them with extra supplements shortly before and during the main discrimination task. In the model (*n* = 68), we used diet (test diet or control diet), age in months and lifelong training score as an independent variable and the interaction of age in months and diet to test whether age effects differed between the diet groups. We also included stimuli (Group A or Group B) and interaction of diet and stimuli, and phase 3 training (4 vs. 4 stimuli or 2 vs. 2 stimuli) as control factors. We included phase 3 training (4 vs. 4 stimuli or 2 vs. 2 stimuli) in the model because the difficulty level of the discrimination task with either 8 or 4 pictures might affect the performance of dogs in the main discrimination task differently.

##### Rate of correction trials

Phase 3: Discrimination training

In this analysis, in addition to dogs that had reached learning criterion, we also included dogs that did not reach criterion but exceeded 1 year of training. In the model for analyzing 4 vs. 4 picture discrimination data (*n* = 31), we included diet (test diet or control diet), age in months, lifelong training score and the interaction of age in months and diet. In the model for analyzing 2 vs. 2 picture discrimination data (*n* = 67), we included diet (test diet or control diet), age in months, lifelong training score, stimuli (Group A or Group B), interaction of age in months and diet, and interaction of diet and stimuli.

Phase 4: Main discrimination task

In addition to dogs that had reached the learning criterion, we also included six dogs that did not reach criterion but had a high number (>22) of sessions. In the model (*n* = 74), we used diet (test diet or control diet), age in months, lifelong training score, stimuli (Group A or Group B), phase 3 training (4 vs. 4 stimuli or 2 vs. 2 stimuli), interaction of age in months and diet, and interaction of diet and stimuli.

## Results

### Discrimination Training (Phase 3: 4 vs. 4 and 2 vs. 2 Discrimination)

In the 4 vs. 4 discrimination task (*n* = 26 dogs), the test diet group took on average 44.1 ± 17.04 sessions and the control diet group took 51.9 ± 23.62 sessions to reach criterion, however, the difference was not significant [estimate = 1.639, *SE* = 0.910, *Z* = 1.801, *p* = 0.071, RR (95% CI) = 5.15 (0.865-2.39), Bayes Factor = 0.128]. We found no interaction effect of age and diet [estimate = -0.013, *SE* = 0.008, *Z* = -1.663, *p* = 0.096, RR (95% CI) = 0.986 (0.971-1.003), BF = 0.400) in regard to the average number of sessions to reach the learning criterion. Also, there was no effect of age [estimate = 0.0072, *SE* = 0.006, *Z* = 1.11, *p* = 0.267, RR (95% CI) = 1.007 (0.995-1.02), BF = 0.047] and lifelong training score [estimate = 0.012, *SE* = 0.009, *Z* = 1.246, *p* = 0.212, RR (95% CI) = 1.012 (0.993-1.031), BF = 0.066] on the average number of sessions needed to reach the learning criterion.

Regarding the rate of correction trials (*n* = 31 dogs), there was no interaction effect of age and diet (estimate = 0.0002, *SE* = 0.001, *Z* = 1.557, *p* = 0.132, $\eta_{p}^{2}$ = 0.085, BF = 0.574), no effect of diet (test diet: 0.26 ± 0.067, control diet: 0.25 ± 0.085; estimate = -0.013, *SE* = 0.028, *Z* = -0.486, *p* = 0.630, $\eta_{p}^{2}$ = 0.008, BF = 0.179), no effect of age (estimate = -0.0004, *SE* = 0.0007, *Z* = -0.622, *p* = 0.538, $\eta_{p}^{2}$ = 0.013, BF = 0.091) and no effect of the lifelong training score (estimate = -0.0003, *SE* = 0.001, *Z* = -0.19, *p* = 0.85, $\eta_{p}^{2}$ = 0.001, BF = 0.079).

In the 2 vs. 2 discrimination task (*n* = 61), the test diet group took on average 16.4 ± 11.31 sessions and the control diet group took 20.9 ± 14.33 sessions, however, the difference was not significant (Table 1). We found no interaction effect of age and diet on the number of sessions to reach the learning criterion and no effect of age. There was no effect of life long training score either on the average number of sessions needed to reach the learning criterion. Finally, there was neither the effect of stimuli nor the interaction with the diet. The calculated Bayes Factors also support these results (see Table 1 for model summary output and BF).

**Table 1:** Results of the generalized linear models and general linear models showing the direction and magnitude of effects (rate ratio or partial eta squared), supplemented with Bayes Factors in Phase 3: 2 vs. 2 picture discrimination task.

Sessions to criteria	Estimate	SE	*Z* value	*p*-Value	Rate_ratio (95% CI)	Bayes Factors
*Age^∗^diet*	0.001	0.006	0.184	0.854	1.001 (0.99, 1.012)	0.148
*Diet*	0.085	0.174	0.487	0.626	1.088 (0.765, 1.527)	0.267
*Age*	0.004	0.003	1.422	0.155	1.004 (0.999, 1.01)	0.052
*Training*	-0.015	0.008	-1.923	0.055	0.985 (0.97, 1)	0.128
*Stimuli*	-0.964	0.182	-5.309	<0.0001*	0.381 (0.268, 0.549)	8660.358
*Diet^∗^stimuli*	0.362	0.254	1.427	0.154	1.436 (0.863, 2.39)	0.308

**Rate of correction trials**					**$\eta_{p}^{2}$**	

*Age^∗^diet*	0.000	0.839	-0.166	0.869	0.000	0.027
*Diet*	0.012	0.020	0.588	0.559	0.013	0.103
*Age*	0.098	0.405	-0.242	0.809	0.001	0.045
*Training*	0.002	0.001	1.622	0.110	0.010	0.137
*Stimuli*	-0.092	0.020	-4.679	<0.00001*	0.252	1681.528
*Diet^∗^stimuli*	0.052	0.039	1.315	0.194	0.028	0.368

^∗^*Means significant*.

Regarding the rate of correction trials (*n* = 67 dogs), we found no interaction effect of age and diet (Table 1). There was no effect of diet (test diet: 0.20 ± 0.099, control diet: 0.21 ± 0.085), age and lifelong training score on the rate of correction trials. The calculated Bayes Factors also support these results (Table 1).

### Main Discrimination Task

**FIGURE 3:**
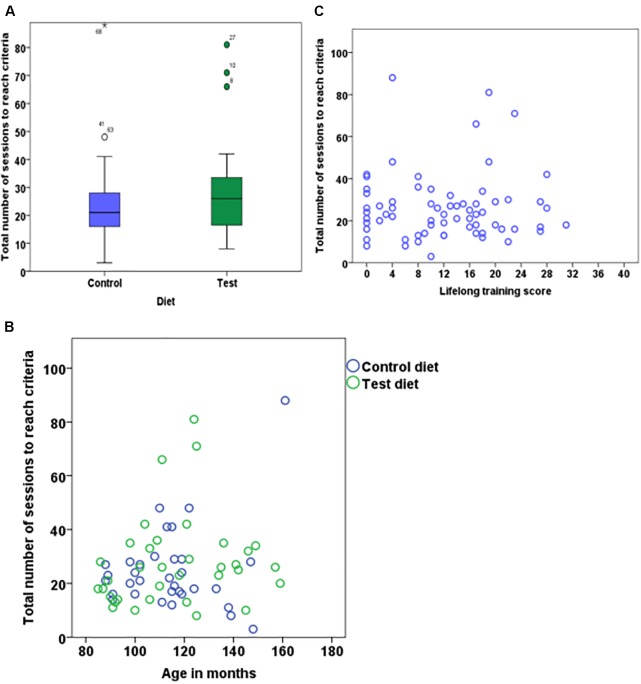
Boxplot showing number of sessions to reach criterion for dogs that received either test diet or control diet **(A)** (*n* = 68, *p* = 0.47). Scatter plot showing the relationship between age in months and number of sessions to reach criterion **(B)** (*n* = 68, *p* = 0.05). Scatter plot showing the relationship between lifelong training score and number of sessions to reach criterion **(C)** (*n* = 68, *p* = 0.54).

On average, dogs on the test diet took 27.7 ± 16.75 sessions (range = 8–81) to reach criterion, while dogs on the control diet needed 24.7 ± 15.41 sessions (range = 3-88); however, the difference was not significant (Figure 3A). Age had neither an effect on the average number of sessions to reach criterion (Figure 3B) nor an interaction with diet (Table 2). Interestingly, there was a significant effect of the phase 3 discrimination training task, with dogs trained on the 4 vs. 4 discrimination task requiring more sessions to reach criterion (31.03 ± 17.83) compared to dogs trained with 2 vs. 2 discrimination task (23.3 ± 14.30) (Table 2). There was no effects of lifelong training score (Figure 3C). Finally, there was neither effect of stimuli nor the interaction with the diet. The calculated Bayes Factor also support these results, except for phase 3 training where the Bayes Factor was 0.542 and therefore gave more support for the absence of an effect while the frequentist inference statistics indicated an effect (see Table 2 for model summary output and BF).

**Table 2:** Results of the generalized linear models and general linear models showing the direction and magnitude of effects (rate ratio or partial eta squared), supplemented with Bayes Factors in the main discrimination task.

Sessions to criteria	Estimate	SE	*Z* value	*p*-Value	Rate_ratio (95% CI)	Bayes Factors
*Age^∗^diet*	0.003	0.006	0.535	0.593	1.003 (0.99, 1.016)	0.172
*Diet*	-0.087	0.123	-0.710	0.478	0.916 (0.721, 1.163)	0.072
*Age*	0.006	0.003	1.959	0.050	1.006 (1, 1.012)	0.126
*Training*	-0.005	0.007	-0.612	0.541	0.995 (0.981, 1.011)	0.333
*Stimuli*	0.164	0.124	1.321	0.187	1.178 (0.931, 1.52)	0.115
*Phase 3 training*	0.288	0.128	2.254	0.024*	1.333 (1.037, 1.706)	0.542
*Diet^∗^stimuli*	-0.004	0.245	-0.014	0.989	0.996 (0.616, 1.586)	0.109

**Rate of correction trials**					**$\eta_{p}^{2}$**	

*Age^∗^diet*	0.000	0.001	0.099	0.921	0.000	0.222
*Diet*	-0.033	0.019	-1.744	0.085	0.041	0.353
*Age*	-0.001	0.000	-1.422	0.159	0.044	0.119
*Training*	0.002	0.001	1.676	0.098	0.059	0.118
*Stimuli*	0.062	0.019	3.189	0.002*	0.124	6.128
*Phase 3 training*	0.000	0.020	-0.014	0.989	0.000	0.089
*Diet^∗^stimuli*	0.000	0.038	-0.003	0.997	0.000	0.170

^∗^*Means significant*.

**FIGURE 4:**
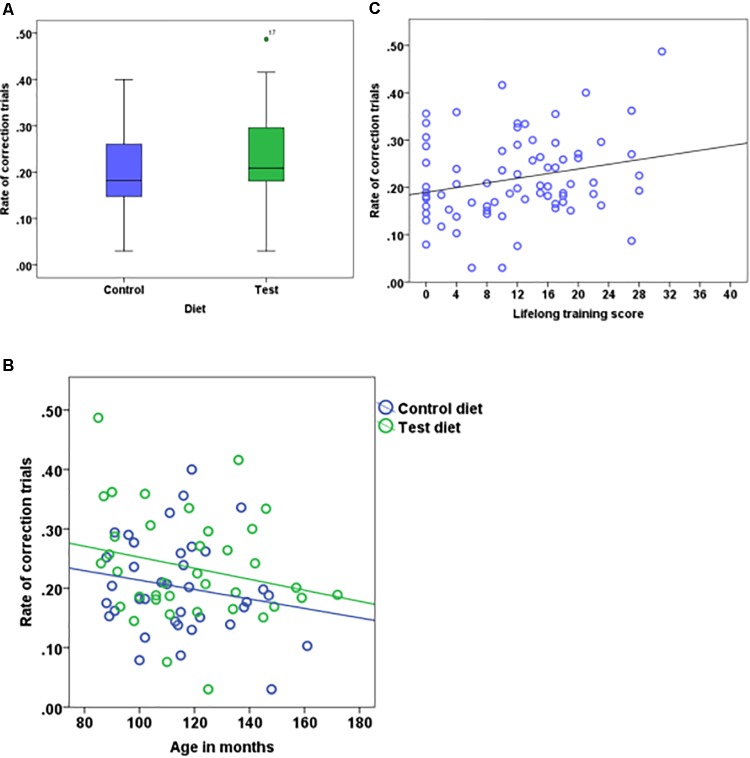
Boxplot showing the rate of correction trials of dogs that received either the test diet or control diet **(A)** (*n* = 74, *p* = 0.08). Scatter plot showing the relationship between age in months and the rate of correction trials **(B)** (*n* = 74, *p* = 0.15). Scatter plot showing the relationship between lifelong training score and the rate of correction trials **(C)** (*n* = 74, *p* = 0.09).

The rate of correction trials also did not differ between the test and control diet groups (test = 0.23 ± 0.092, control = 0.20 ± 0.083, Figure 4A). We found no interaction effect of age and diet (Table 2). The rate of correction trials for dogs in Group B stimuli category (0.25 ± 0.083) was significantly higher compared to dogs in Group A (0.18 ± 0.085). There was no effect of age (Figure 4B), lifelong training score (Figure 4C) and phase 3 discrimination training (2 vs. 2 = 0.21 ± 0.087, 4 vs. 4 = 0.22 ± 0.09). The calculated Bayes Factors also support these results (Table 2).

## Discussion and Conclusion

In this study, we examined the changes in discrimination learning in older pet dogs of various breeds using a procedure that had previously been shown to reveal age effects and tested the effectiveness of lifelong training as well as a dietary enrichment with antioxidants (vitamin C, vitamin E, and polyphenols), DHA, phosphatidylserine, and tryptophan in counteracting a decline in learning. The results showed no effect of age, lifelong training and diet on the speed of learning determined by the number of sessions to reach criterion, and deficit in learning from feedback shown by the rate of correction trials.

It is difficult to compare our study results with other studies examining age effects on discrimination learning due to differences in the ages of the subjects used in these studies. Studies reporting age effects in discrimination learning mostly included younger dogs in their sample, which might then lead to significant effects when compared to older dogs (Milgram et al., 2002a,b; Tapp et al., 2003a; Wallis et al., 2016). Nevertheless, given that Wallis et al. (2016) found a linear age effect, we would have expected to see a decline in performance in our dogs with increasing age despite the fact that we only tested older dogs, which was not the case in Wallis study. This might be explained by several differences between the two studies. First, Wallis et al. (2016) compared 81 younger dogs (<6 years) with 14 older dogs (>6 years) which is different to our study sample that included only dogs over 6 years of age. In Wallis et al. (2016) study, dogs aged from 5 months to 1 year took the lowest number of sessions to reach criterion compared to all age groups over 1 year, highlighting that this age group was already performing at the peak level in discrimination learning. All dogs over 3 years took more sessions to reach the learning criterion, demonstrating that the learning abilities begin to decline early during life. However, this cannot be the sole explanation since, when we reanalyzed the data from Wallis et al. (2016) but restricted the analysis to only older dogs (>6 years; *n* = 14), we still found a linear increase with age in their Border collie sample. What might have contributed to this difference between the two studies is that our discrimination training (phase 3) was more similar to our main discrimination task (phase 4) than in Wallis et al. (2016) study. It has been shown that carryover learning effects from previous training affect the performance of dogs in discrimination learning (Milgram et al., 2002a). Even more, we found that the dogs that had received discrimination training, similar to the main discrimination task with 2 vs. 2 stimuli performed better in the main discrimination task compared to the dogs trained with 4 vs. 4 stimuli, suggesting that the similarity between training and main task matters. Therefore, previous training on 2 vs. 2 stimuli might have also impacted the performance of dogs on the main discrimination task after 1 year. However, we should be cautious with this interpretation since the effect of phase 3 training was not supported by the Bayesian statistics.

A second possible explanation is the difficulty of the task. Previous studies in humans (Boutet et al., 2005), non-human primates (Rapp, 1990; Bachevalier et al., 1991; Lai et al., 1995) and laboratory dogs (for review, see Davis and Head, 2014) have found no effect of aging on associative learning in simple object discrimination tasks. However, age effects were more visible when the discrimination task was difficult (Rapp, 1990; Voytko, 1999; Milgram, 2003; Snigdha et al., 2016). By utilizing a higher number of stimuli (four positive and four negative) to be discriminated, Wallis et al. (2016) might have sufficiently increased the difficulty level to allow for an age effect to emerge. Dogs in our study had to discriminate two positive and two negative stimuli, and, in addition, the criterion set to learn the discrimination in our study (≥27 correct in two consecutive sessions) was lower than in Wallis et al. (2016) (≥28 correct in five consecutive sessions). These two factors might have reduced the difficulty of the task. However, we purposefully reduced the number of stimuli to be discriminated and also lowered the criteria of learning to enable all dogs, even the oldest ones, to successfully complete the task. Moreover, we found no age effect in the discrimination training (phase 3) either when, for 26 dogs, we used the same discrimination task (four positive and four negative) as Wallis et al. (2016). This suggests that task difficulty in itself cannot explain the lack of age effects in our sample.

A third possible explanation for the difference between the results in the two studies is that while Wallis et al. (2016) tested only Border collies, we included 30 different breeds in our sample. Breed differences in trainability has been documented in dogs (Helton, 2010) with Border Collies at the top end. Since trainability probably goes together with learning ability, we can consider that Border collies in general are better at learning. Therefore, age effects might actually be more profound and more easily detectable in Border collies.

Similar reasons proposed above can also explain why we did not find age effects on the rate of correction trials either which is contrary to many studies in humans and dogs (Milgram et al., 2002a; Tapp et al., 2003a; Boutet et al., 2005; Snigdha et al., 2012; Wallis et al., 2016).

Even if we found no age effect in our sample, an effect of the enriched diet could have occurred. The majority of studies reporting diet effects in laboratory dogs have documented age effects in samples that contained also dogs with cognitive decline. Even more, it has been shown that antioxidants are more effective in older dogs with cognitive decline as compared to younger dogs with intact cognition (Milgram et al., 2004, 2005). Our subjects were relatively younger (6 years, with 34.2% of the dogs younger than 8 years of age) than the dogs in the majority of these studies that used older dogs (>8 years) to test diet effects. Nevertheless, there are studies in laboratory dogs that have shown that older laboratory dogs (>8 years) on an antioxidant diet committed fewer errors compared to dogs on a control diet in a landmark discrimination task (Milgram et al., 2002a), size discrimination (Milgram et al., 2004), oddity discrimination task (Milgram et al., 2002b), and in a visual discrimination and reversal task (Milgram et al., 2005). However, recently, a study in laboratory dogs has demonstrated that these enriched diets with antioxidants are not always effective (Snigdha et al., 2016). Moreover, in humans, a recent review by Forbes et al. (2015) has provided strong evidence of no effect of omega-3 fatty acids and vitamin E on the outcomes of cognitive task. Lately, several studies have highlighted that enriched diets formulated to counteract aging do not have any effects on cognition. Nevertheless, the media hype in advertising the anti-aging diets and products makes them more visible to the consumers regardless of its effectiveness (MacGregor et al., 2018).

In our study, the lack of diet effect may be due to the diet being ineffective or other various factors that differ between pet dogs and laboratory dogs with the latter being mostly genetically homogenous and living in identical and non-stimulating environments. Pet dogs in contrast live in variable and stimulating environments. They may share their life with a single owner or with several people in the household, and they may need to adjust to living in a multi-dog household where they need to compete or cooperate with other dogs for food or for attention. They are physically as well as mentally challenged in their home but also outside, during walks or when meeting familiar and unfamiliar people and dogs in various circumstances, and they often receive training of many different forms. Rearing animals in enriched and stimulating environments has been reported to improve learning ability, to produce beneficial changes in cellular structure and to increase the resistance of neurons to injury (Mattson et al., 2001). Moreover, physical activity is associated with improved cognitive function and lower risks of cognitive impairment and dementia in humans (Cotman and Berchtold, 2002). It is plausible that the enriched and stimulating environment of pet dogs slow down their aging process as compared to laboratory dogs. Hence, if we find no effect of age on their learning and cognitive flexibility, any counter- acting effect of the diet on aging cannot be detected.

It is also possible that the feeding duration was not long enough to see an effect of enriched diet. There are contradictory findings in regard to the length of feeding needed to detect effects of dietary treatments. Similarly to our results, despite feeding older laboratory dogs with enriched diet for 6 months, Head et al. (2012) found no effect of diet on oddity and size discrimination learning. Milgram et al. (2005) also emphasized that the effect of an enriched diet on cognition is more robust after 2 years on diet than after only 1 year. However, other studies conducted on laboratory dogs have shown positive effect of enriched diet with antioxidants and mitochondrial co-factors already after feeding for 3 months (Milgram et al., 2002a), 6 months (Milgram et al., 2002b) and 1 year (Milgram et al., 2004). Additionally, there are two studies in pet dogs (Dodd et al., 2003; Heath et al., 2007) that have shown beneficial effects of nutritional supplement as early as 2 months of feeding. Noteworthy, these studies, however, relied completely on the owners’ assessment of dogs’ behavioral changes and used no objective measurement of cognitive functions with specific tests. Therefore, it is difficult to make any strong conclusion regarding the effectiveness of enriched diets in hindering cognitive aging in pet dogs.

Finally, it is possible that the baseline nutrient level of two groups were not equivalent. Since we did not measure blood parameters regarding the concentration of different nutrients at baseline, we are not sure whether the effect of nutrients got dissolved in the test diet group since the baseline values for this group might be lower compared to the control group. So the nutritional benefit that they got through the diet did not show up. Or we could speculate that the control diet used in our study was already so healthy, i.e., fulfilling the bodily needs of antioxidants, DHA, Phosphatidylserine and tryptophan that there is simply no additional benefit possible from the supplementation with the enriched diet. Dogs participating in our study might be already eating healthy diet before they got enrolled for the study and therefore switching to the study diets might not have changed much in their nutritive gain which can explain why we did not find diet effect in our study.

In the current study, we found no effect of lifelong training on the dogs’ learning performance. In humans, there are a number of studies that support the beneficial effects of training, both in-task training and lifelong training experiences on the physical and cognitive health of older humans, assessed by measuring different cognitive functions (Churchill et al., 2002; Colcombe et al., 2003; Chang et al., 2012). Studies in humans, rats and laboratory beagles have also documented that environmental enrichment, cognitive training and physical activity can improve cognitive performance (Milgram et al., 2005; Kramer et al., 2006; Nippak et al., 2006; Berchtold and Cotman, 2009; Snigdha et al., 2014). There is some evidence for a positive effect of lifelong training on dogs’ problem solving abilities (Marshall-Pescini et al., 2008, 2009, 2016). However, so far the issue of whether cognitive training can improve performance within cognitive domains not trained (i.e., far transfer) has not been examined in dogs. In humans, it is well-established that training a specific cognitive ability results in improvements in that task (i.e., practice effect) and generally in similar tasks (i.e., near transfer). However, evidence regarding whether training a particular cognitive domain transfers to improvement across other untrained cognitive tasks (i.e., far transfer) is mixed (Goghari and Lawlor-Savage, 2017). In line with this, Goghari and Lawlor-Savage (2017) did not find an effect of 8 weeks of computerized cognitive training on the performance of humans in working memory, planning, reasoning, processing speed, verbal fluency, cognitive flexibility and creativity tasks. Accordingly, Mosing et al. (2016) also did not find far transfer effects of music practice on general cognitive ability in humans. In our previous study, we found that dogs with more lifelong training experiences performed better in different attention measures (Chapagain et al., 2017). Noteworthy, in the majority of training tasks, attention is the most crucial aspect since the prerequisite for these tasks is that the dog is attentive to the owner/trainer/handler and to the environment. Therefore, in the previous study, the dogs that had a higher lifelong training score might have been able to retain the attention in the two tasks that we used to assess sustained and selective attention better due to their training, especially in attentiveness than dogs that had not so much experience. It seems plausible to speculate that near transfer of attention skills made the old dogs capable of retaining different attention measures. Nevertheless, in picture discrimination task, in addition to attention, working memory is also involved and thus the effect of far transfer may not be associated with the degree of lifelong training.

In conclusion, this study showed no difference in the speed of learning in pet dogs of older ages (>6 years) and no effect on cognitive flexibility either. Also, the benefit of an enriched diet with antioxidants, DHA, phosphatidylserine and tryptophan on the learning ability of older dogs using a discrimination learning task could not be confirmed. This is likely partly due to our experimental design where training the dogs in a task similar to the main discrimination task might have masked the existing age and diet effects. The absence of an age effects in the task made it highly unlikely to find any diet effects. Accordingly, there might be a diet effect that we could not detect. Therefore, future studies should improve the training protocol so that dogs are trained in a simpler task during the baseline and the complexity of task should increase during the main discrimination test. Moreover, it seems more plausible to use dogs older than 8 years to find actual cognitive decline, and speculate that we would find an effect of diet given different circumstances. As our study not only failed to detect the expected effects, our results support the null hypothesis. Hence, more work is necessary to assess the effect of age, diet and lifelong training on the performance of older pet dogs in discrimination learning tasks.

## Author Contributions

DC, ZV, and FR conceived and designed the experiments. DC and JuS performed the experiments. DC, ZV, and FR analyzed the data. DC, ZV, FR, LH, and JeS interpreted the results. DC prepared the first draft of manuscript. DC, ZV, FR, LH, and JeS revised the paper.

## Conflict of Interest Statement

The authors declare that the research was conducted in the absence of any commercial or financial relationships that could be construed as a potential conflict of interest.

## Footnotes

1 https://www.akc.org/

2 https://stefany.shinyapps.io/RcountD/
