# Supplementary material for: Effect of Age and Dietary Intervention on Discrimination Learning in Pet Dogs
Source: Front Psychol. 2018 Nov 14;9:2217. doi: 10.3389/fpsyg.2018.02217 (PMC6246696; doi:10.3389/fpsyg.2018.02217)
Supplement: Supplementary file 1 [file Data_Sheet_1.pdf]

Supplementary material

Subjects:

Table S1: Individual characteristics of dogs that participated in the final picture discrimination task in the touchscreen after one year of dietary intervention**.**

No.	Name	Age (yrs.)	Sex	Breed	Weight	Training score	Diet	Stimulus _group	Status
**1**	Fynn	7.3	M	Poodle	9.5	17	test	GroupA	Finished
**2**	Apryl	7.5	F	Border Collie	18	14	test	GroupA	Finished
**3**	Taiko	7.8	M	American Akita	33.7	12	test	GroupA	Finished
**4**	Fenja	7.9	F	Mixed	10	9	test	GroupA	Finished
**5**	Kimba	8.2	F	Mixed	25	0	test	GroupA	Finished
**6**	Leila	8.4	F	Mixed	20	22	test	GroupA	
**7**	Shane	8.1 0	F	Belgian shepherd	22	18	test	GroupA	Finished
**8**	Forin	9.2	F	Border Collie	16	12	test	GroupA	Finished
**9**	Nemo	9.3	M	Labrador	35.5	17	test	GroupA	Finished
**10**	Guinness	10.5	F	Border Collie	20	NA	test	GroupA	Finished
**11**	Nash	10.5	M	German Shepherd	34.2	23	test	GroupA	Finished
**12**	Geena	11.2	F	Border Collie	19	17	test	GroupA	Finished
**13**	Shadow	11.4	M	Mixed	15.9	10	test	GroupA	Finished
**14**	Wichtel	11.9	M	Pyrenean Shepherd	11	14	test	GroupA	Finished
**15**	Jack	12.1	M	Mixed	23.3	8	test	GroupA	Finished
**16**	Enigma	12.5	F	Border Collie	20.3	18	test	GroupA	Finished
**17**	Trisha	13.3	F	Border Collie	25	2	test	GroupA	Finished
**18**	Bosco	14.4	M	Mixed	32.4	0	test	GroupA	Did not finish but data used for correction trials
**19**	Flamme	7.1	M	Pyrenean Shepherd	18	31	test	GroupB	Finished
**20**	Amadeus	7.2	M	Cavalier King Charles	10	17	test	GroupB	Finished

				Spaniel					
**21**	Marlo	7.6	M	Australian Shepherd	25	27	test	GroupB	Finished
**22**	Hybie	7.7	F	Labrador	28.3	0	test	GroupB	Finished
**23**	Riga	8.6	F	Entelbucher Mountain dog	18	4	test	GroupB	Finished
**24**	Akira1	8.7		American staff	30	0	test	GroupB	Finished
**25**	Frankie	8.8	M	Golden Retriever	32.1	0	test	GroupB	Finished
**26**	Scully	9.1	F	Mixed	28.7	8	test	GroupB	Finished
**27**	Jacky	9.3	M	Mixed	13.5	11	test	GroupB	Finished
**28**	Flora	9.1 0	F	Golden Retriever	37.3	12	test	GroupB	Finished
**29**	Bastian	10.1	M	Border Collie	25	8	test	GroupB	Finished
**30**	Quinnie	10.1	F	Malinois	28.7	28	test	GroupB	Finished
**31**	Akita	10.2	F	Mixed	21	20	test	GroupB	Finished
**32**	Ginger	10.4	F	Parson Russel Terrier	10.1	19	test	GroupB	Finished
**33**	Darwin	11	M	Border Collie	23.2	15	test	GroupB	Did not finish but data used for correction trials
**34**	Flori	11.3	F	Mixed	17	28	test	GroupB	Finished
**35**	Tilly	11.1 0	F	Parson Russel Terrier	7	16	test	GroupB	Finished
**36**	Ilvy	12.2	F	Wolfspitz	20.3	13	test	GroupB	Finished
**37**	Kora	13.1	F	Mixed	23	0	test	GroupB	Finished
**38**	Sue	7.4	F	Border Collie	19	13	control	GroupA	Finished
**39**	Leopold	7.7	M	Mixed	9	17	control	GroupA	Finished
**40**	Missface	8.2	F	Mixed	22	10	control	GroupA	Finished
**41**	Patrasch	8.4	M	Mixed	15	0	control	GroupA	Finished
**42**	Rocky_mix	8.6	M	Mixed	17	2	control	GroupA	Finished
**43**	Cookie	9.2	F	Mixed	36	4	control	GroupA	Finished
**44**	Havanna	9.5	F	Beagle	10	8	control	GroupA	Finished
**45**	Ella	9.6	F	Münsterländer	25	4	control	GroupA	Finished
**46**	Luke	9.7	M	Border Collie	24.3	27	control	GroupA	Finished
**47**	Nora	9.7	F	Mixed	20	18	control	GroupA	Finished
**48**	Timi	9.1 0	F	Magyar Viszla	20	16	control	GroupA	Finished

**49**	Sayzi	9.11	M	Mixed	0	17	control	GroupA	Finished
**50**	Ginger4	11.1	F	Mixed	9.1	10	control	GroupA	Finished
**51**	Isla	11.6	F	Podenco	19	6	control	GroupA	Finished
**52**	Sunny	12.3	M	Border Collie	20	15	control	GroupA	Finished
**53**	Todor	12.4	M	Mixed	15	NA	control	GroupA	Finished
**54**	Elvin	7.4	M	Mixed	9	0	control	GroupB	Finished
**55**	Elina	7.5	F	Mixed	18	3	control	GroupB	Finished
**56**	Gismo	7.7	M	Border Collie	23.7	23	control	GroupB	Finished
**57**	Jersey	8.2	F	Magyar Viszla	22	10	control	GroupB	Finished
**58**	Tika	8.4	F	Mixed	28.8	18	control	GroupB	Finished
**59**	Nova	8.6	F	Mixed	22.4	16	control	GroupB	Finished
**60**	Socrates	9	M	Mixed	31.4	22	control	GroupB	
**61**	Gwendolyn	9.3	F	White Swiss shepherd	34	12	control	GroupB	Finished
**62**	Chelsey	9.7	F	Mixed	42	0	control	GroupB	Finished
**63**	Queenie	9.8	F	Beagle	13.1	4	control	GroupB	Finished
**64**	Ronja	9.8	F	Parson Russel Terrier	8	0	control	GroupB	Finished
**65**	Akira_mix	9.11	F	Mixed	23	21	control	GroupB	Finished
**66**	Chil	9.11	F	Australian Shepherd	16	27	control	GroupB	Finished
**67**	Sidney	10.2	M	Border Collie	23	19	control	GroupB	Finished
**68**	Mago	10.4	M	Golden Retriever	25.5	20	control	GroupB	Finished
**69**	Habibi	11.6	F	Mixed	16	0	control	GroupB	Finished
**70**	Poris	13.5		Mixed	9.5	4	control	GroupB	Finished
**71**	Kiwi	7.9	F	Mudi	10	0	control	GroupB	Finished but data discarded due to feeding suppleme nts
**72**	Haeger	11.4	M	Australian Shepherd	22.4	30	control	GroupA	Finished but data discarded due to feeding suppleme nts
**73**	Mozart	10	M	Golden Retriever	31.1	13	test	GroupB	Finished but data discarded

									due to feeding suppleme nts
**74**	Rocky	8	M	Labrador Retriever	29	12	control		Did not finish but data used for correction trials
**75**	Porthos	11.5		Mixed			control	GroupB	Did not finish but data used for correction trials
**76**	Chilli	12.4	F	Herder	24	34	control	GroupB	Did not finish
**77**	Nanouk	7.6	M	Australian Shepherd	21.1	15	control	GroupB	Did not finish but data used for correction trials
**78**	Toby	12.1	M	Labrador Retriever	27	12	control	GroupA	Did not finish but data used for correction trials
**79**	Fipsi	13.1	M	Mixed	10.7	10	control	GroupB	Did not finish

Methodology:

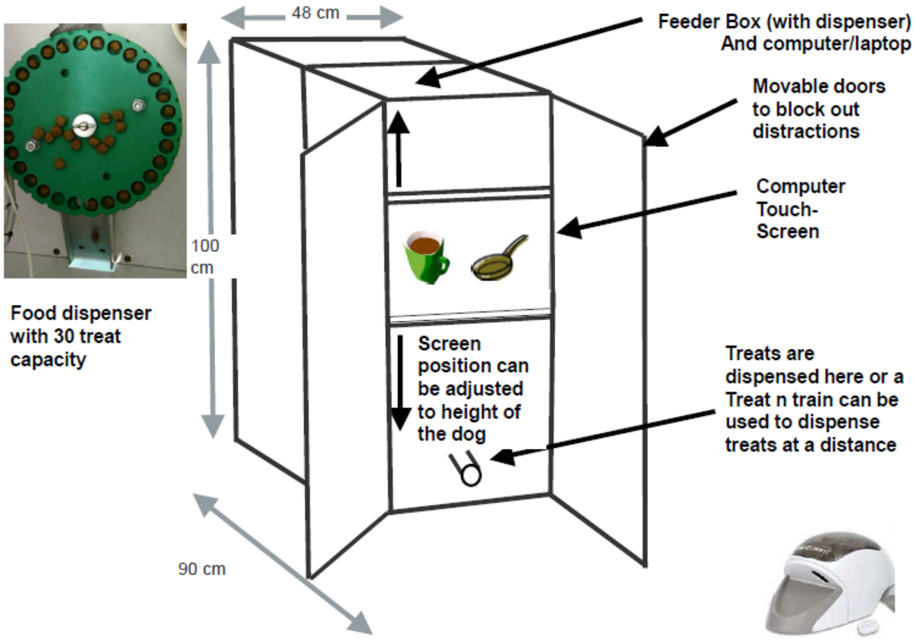

Figure S1. Schematic diagram of the touchscreen apparatus, including the feeder box (containing food dispenser and computer), movable doors to block out distractions and adjustable touchscreen monitor. Treats were dispensed through a tube from the feeder box, or through a feeding device (Treat & Train) to dispense treats at a distance. Top left: Photograph of the food dispenser inside the touchscreen apparatus. Bottom right: Treat & Train automatic food dispenser with remote control. This diagram is adapted from Wallis et al. (2017).

1. **Phase 2: Discrimination pre-training (Geometric form discrimination or picture discrimination (1 vs 1))**

Fourty five dogs (23 dogs on test diet and 22 dogs on control diet) participated in the geometric form discrimination**.** In this task, the dogs were shown a yellow triangle and a yellow arrow side by side on the screen of the touchscreen. Half of the dogs in each diet group were randomly allocated to Group 'triangle' and other half were allocated to Group "arrow". Group "triangle" was rewarded for touching the triangle; group 'arrow' was rewarded for touching the arrow.

Seventy dogs (35 dogs on each of two diets) participated in the picture discrimination training with two pictures (1 vs 1). In this task, the subjects were shown two clip art pictures side by side on the screen of touchscreen. The stimuli differed in colour, global outline, and internal features. This allowed the dog to more easily discriminate them. Half of the dogs in each diet group were randomly allocated to the Group 'basket' and the other half were allocated to Group "bowl".

Group “basket” was rewarded for touching the basket; group ‘bowl’ was rewarded for touching the bowl. The presentation of the stimuli on the touchscreen, the training procedure and learning criteria were identical in both versions of the discrimination pre-training. Since the dogs only learned to touch one stimulus in the approach training, some dogs that found it difficult to learn this task were often helped verbally or in some occasions the experimenter pointed to the correct stimulus.

Results

Phase 2: Discrimination pre-training

In the geometric form discrimination, 41 out of 45 dogs completed the training while 67 out of 70 dogs that participated in the picture discrimination task completed the training. Four dogs of the geometric form discrimination and 3 dogs presented with the picture discrimination task did not reach the criterion because of lack of motivation to work on the touchscreen.

In the geometric form discrimination, dogs on the test diet (n=19) took on average 12.57 ± 7.12 sessions (range=4-28) to reach criterion, while dogs on the control diet (n=22) needed 10.63 ± 6.88 sessions (range=4-26). In the 1x1 picture discrimination task, dogs on the test diet (n=32) took 13.85 ± 8.15 sessions (range=4-40) to reach criterion, while dogs on the control diet (n=35) needed 13.34 ± 8.68 sessions (range=4-36).

Phase 3: Discrimination training (Phase 3: 4 vs. 4 and 2 vs. 2 discrimination)

Of the 34 dogs that started with the 4 vs. 4 training, only 26 passed the learning criterion. When they started discrimination training, dogs in the test group (n=11) had been on diet for 167.6 ± 65.6 days (mean ± SD) whereas control dogs (n=15) for 140.7 ± 75.8 days. Five dogs that did not pass the learning criterion exceeded one year of training period and therefore their training was stopped. Three other dogs dropped from the training because the owners terminated the training (n=2) and due to health problems (n=1). Of the 72 dogs that started with the 2 vs. 2 training, 61 dogs passed the criterion. When they started discrimination training, test dogs (n=31) had been on diet for 88.7 ± 98.1 days whereas control dogs (n=30) for 102.4 ± 80.1 days. Eleven dogs did not pass the learning criterion because their owners quitted the training (n=5) or they exceeded one year of training period (n=6).

Dogs trained on 4 vs. 4 stimuli took more sessions to reach the learning criterion (48.9 ± 21.01 sessions) compared to dogs trained on 2 vs. 2 stimuli (18.6 ± 12.97 sessions; estimate= 1.03, SE=0.13, Z= 7.53, $p<0.001$).

Phase 4: Main discrimination task

Among 79 dogs that participated in the main task, 71 dogs (n=36 dogs on test diet, n=35 control dogs) reached the learning criterion. However, 3 dogs had to be excluded from the analyses as their owners fed them with extra supplements shortly before and during the main discrimination

task. The 68 dogs included in the analyses needed 3 to 88 sessions to reach criterion. Eight dogs (test diet: 2 and control diet: 6) that did not reach the learning criterion participated in 82, 78, 55, 32, 27, 22, 8 and 3 sessions respectively.

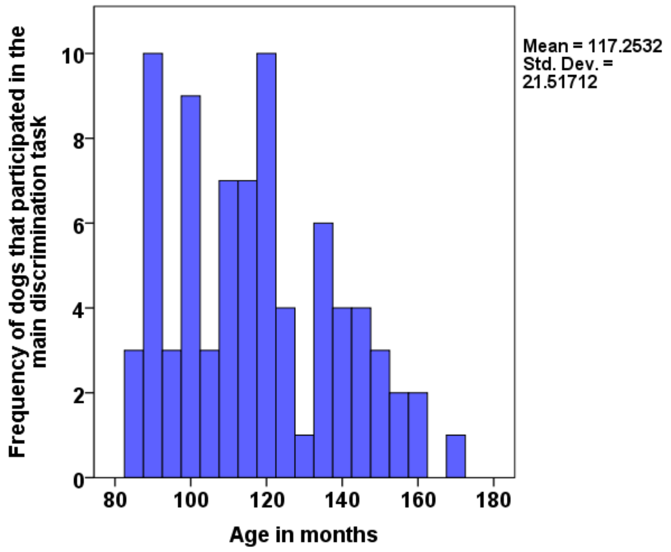

Fig. 2. Histogram showing the distribution of age in 79 dogs that participated in the main discrimination task.

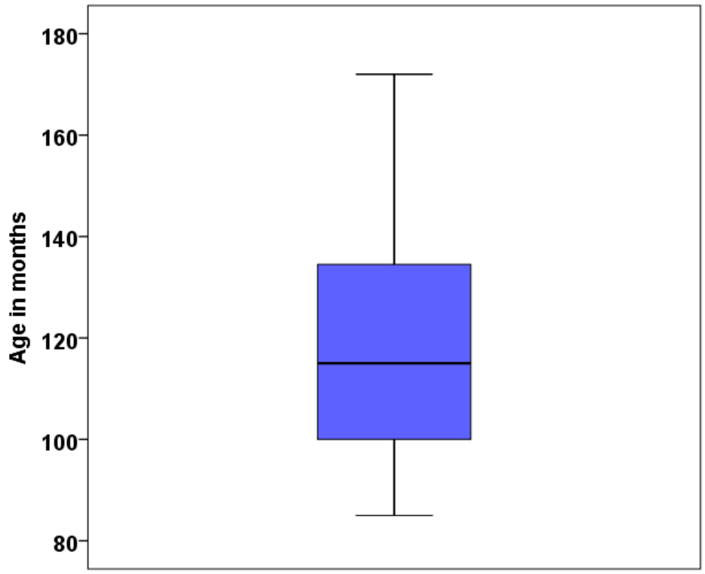

Fig. 2. Boxplot showing the distribution of age in 79 dogs that participated in the main discrimination task. The segment inside the rectangle shows the median age of subjects and the wisker above the box shows the maximum age of subjects and the wisker below the box shows the minimum age of subjects.

References:

Wallis, L. J., Range, F., Kubinyi, E., Chapagain, D., Serra, J., Huber, L. 2017. Utilising dog-computer interactions to provide mental stimulation in dogs especially during ageing. ACI2017. Proceedings of the Fourth International Conference on Animal-Computer Interaction Article No. 1. Doi >10.1145/3152130.3152146
